# Chemo-Strain Valence Engineering for Boosting Photovoltaic Response in Double Perovskite Epitaxial Films

**DOI:** 10.1007/s40820-026-02105-y

**Published:** 2026-03-04

**Authors:** Yonghui Wu, Jie Tu, Jing Xia, Xudong Liu, Longyuan Shi, Hangren Li, Menglin Li, Peng Chen, Qianqian Yang, Siyuan Du, Pengfei Song, Haiying Li, Qian Zhan, Xiaolong Li, Jianjun Tian, Linxing Zhang

**Affiliations:** 1 Institute for Advanced Materials Technology, University of Science and Technology Beijing, Beijing, 100083 People’s Republic of China; 2 Key Laboratory of Photochemical Conversion and Optoelectronic Materials, Technical Institute of Physics and Chemistry, Chinese Academy of Sciences, Beijing, 100190 People’s Republic of China; 3 School of Electronic Science and Engineering, Collaborative Innovation Center of Advanced Microstructures, Nanjing University, Nanjing, 210093 People’s Republic of China; 4 School of Materials Science and Engineering, University of Science and Technology Beijing, Beijing, 100083 People’s Republic of China; 5 Shanghai Synchrotron Radiation Facility, Shanghai Advanced Research Institute, Chinese Academy of Sciences, Shanghai, 201204 People’s Republic of China; 6 Institute of Solid State Chemistry, University of Science and Technology, Beijing, Beijing, 100083 People’s Republic of China

**Keywords:** Chemical strain, Defect engineering, BiFeO_3_-based, Ferroelectric photovoltaics, Double perovskite

## Abstract

Double perovskite films offer significant potential for multiferroic and ferroelectric photovoltaics due to their structural tunability. This study employs an aliovalent substitution strategy, partially replacing Bi with Pb in Bi_2_FeMnO_6_ (BFMO), to disrupt charge balance and local polarization while maintaining the host lattice. Pb incorporation simultaneously modulates the chemical states of all constituent elements, inducing pronounced lattice distortion and positive chemical strain. Unpoled Pb-BFMO films exhibit exceptional photovoltaic performance under 80 mW cm^−2^ illumination, achieving a short-circuit current density (|*J*_SC_|) of 192 μA cm^−2^ and an open-circuit voltage (|*V*_OC_|) of 0.525 V. This represents a 109-fold increase in intrinsic *J*_SC_ and a fourfold enhancement in V_OC_ compared to pure BFMO. The |*J*_SC_| demonstrates electric field tunability via polarization switching, reaching 320 μA cm^−2^ under negative polarization, the highest reported *J*_SC_ for sub-100 nm single-layer ferroelectric films under white light. High-resolution high-angle annular dark-field scanning transmission electron microscopy, synchrotron-based reciprocal space mapping and X-ray absorption spectroscopy analyses collectively confirm the coupling of crystal distortion, chemical strain, and valence state alterations. The synergy between chemical strain and ionic valence states effectively engineers the bandgap and enhances photovoltaic response, which unlocks new application pathways for perovskite materials in optical memory devices and sustainable energy systems.

**Supplementary Information:**

The online version contains supplementary material available at 10.1007/s40820-026-02105-y.

## Highlights

- A simple inequivalent substitution strategy modulates lattice distortion and element valence states, realizing a remarkable boost in ferroelectric photovoltaic performance under white light (|*J*_SC_| = 320 μA cm^−2^ after negative poling).
- Synergistic integration of chemical strain and defect engineering yields high performance ferroelectric photovoltaic in double perovskite thin films.
- Oxygen vacancies enable electric-field modulation of photovoltaic response in ferroelectric thin films by tuning the band gap and engineering the Schottky barrier.

**Supplementary Information:**

The online version contains supplementary material available at 10.1007/s40820-026-02105-y.

## Graphical abstract

**Figure:**
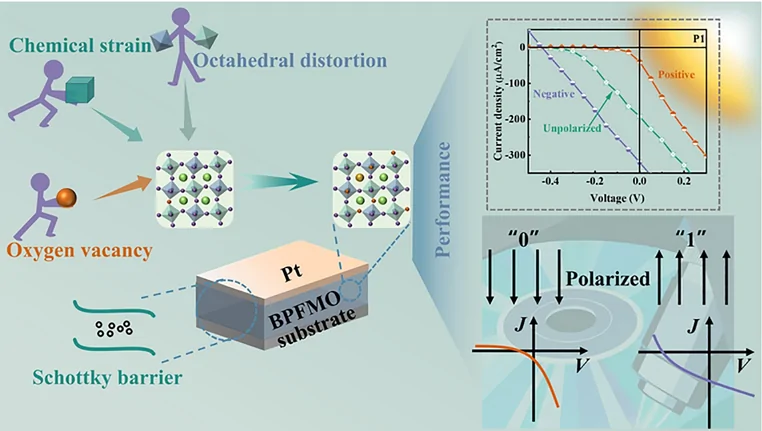

## Introduction

Significant research attention has recently been directed toward ferroelectric photovoltaics due to their fundamentally distinct carrier-generation mechanisms compared to conventional semiconductor p-n junctions [1]. Consequently, anomalously high photovoltages have been observed in thin-film ferroelectrics such as Pb(Zr, Ti)O_3_ [2–4], BaTiO_3_ [5, 6], and BiFeO_3_ [7, 8], with photocurrent directionality being demonstrated to be reversibly controlled through polarization switching. However, the practical deployment of traditional ferroelectric photovoltaics has been constrained by inherent material limitations: wide bandgaps (> 2.5 eV) and high electrical insulation result in low visible-light absorption efficiencies and suppressed carrier mobilities, respectively. Although BiFeO_3_ (BFO) initially attracted interest for its room-temperature multiferroicity, its photovoltaic potential was later recognized following the discovery of electrically tunable diode effects [7] and subsequent investigations of its bulk photovoltaic properties [8, 9]. With a reduced bandgap (~ 2.6 eV) [10, 11], BFO has emerged as a promising candidate for ferroelectric photovoltaics. Nevertheless, its optical absorption capacity remains substantially inferior to that of mainstream semiconductors (e.g., Si, Ge) [12], limiting practical photocurrent generation.

Double perovskite oxide films offer exceptional compositional tunability and highly controllable electronic structures, providing a versatile platform for tailoring functional properties [13]. This inherent flexibility allows for strategic adjustments of key parameters, which frequently induce significant structural modifications and enable precise regulation of diverse physical functionalities [14]. A notable advancement in this field was the achievement of an 8.1% photoelectric conversion efficiency under AM1.5G illumination in Bi_2_FeCrO_6_ (BFCO) films, realized through B-site cation ordering engineering. This breakthrough vividly demonstrated the substantial application potential of double perovskites within the domain of ferroelectric photovoltaics [15, 16]. Research on BFCO photovoltaic mechanisms has progressively deepened, establishing a critical link between the degree of Fe/Cr ordering and the material's bandgap through modification of deposition conditions—a tunability inherent to the double B-site element characteristic of these perovskites [17]. Further theoretical insights emerged from first-principles analyses, proposing that effective electron–hole separation localized on the B-site elements underpins the significant ferroelectric photovoltaic effect observed in BFCO [18]. Beyond the extensively studied BFCO systems, exploration of alternative double perovskite compositions has revealed distinct advantages for ferroelectric photovoltaics. Recent work on Bi_0.5_Sm_0.5_FeO_3_ (BSFO) thin films, synthesized via magnetron sputtering, has provided comprehensive insights into their crystal structure, coupled ferroelectric–ferromagnetic properties, and intrinsic ferroelectric photovoltaics response [19]. Crucially, the application of oxygen vacancy engineering to BSFO emerged as a potent strategy, effectively modifying its structure and enabling precise bandgap tuning [20]. This deliberate manipulation yielded a remarkable enhancement in device performance; the *V*_OC_ in Pt/BSFO/Nb-SrTiO_3_ (NSTO) structures surged from 0.50 to 1.56 V, demonstrating the significant impact of defect control. The scope of promising ferroelectric photovoltaics materials continues to broaden, encompassing novel double perovskites such as CaMnTi_2_O_6_ [21] and chemically tuned Bi_2_FeMo_*x*_Ni_(1-*x*)_O_6_ [22]. Collectively, these advances underscore the considerable application potential of double perovskite materials in ferroelectric photovoltaics.

Mn has been identified as another promising B-site candidate for BFO-based double perovskite systems of Bi_2_FeMnO_6_ (BFMO) [16]. Although previous theoretical models have demonstrated that BFMO is a promising ferroelectric photovoltaic material [16, 18], initial synthesis and foundational studies of BFMO thin films were reported over a decade ago, encompassing both experimental and theoretical investigations [23]. However, subsequent research largely concentrated on elucidating BFMO's crystal structure, multiferroic behavior, and electronic transport properties [24–28]. This significant focus left a pronounced gap in the fundamental understanding and systematic characterization of BFMO's ferroelectric photovoltaic performance. It was not until recently that this critical knowledge gap began to be addressed. A breakthrough occurred with the successful preparation of BFMO films via pulsed laser deposition (PLD), enabling the first comprehensive characterization of their ferroelectric photovoltaic properties [29]. This seminal work demonstrated a dramatic enhancement in photovoltaic performance compared to the prototypical BiFeO_3_ (BFO), the short-circuit current density (*J*_SC_) increased by two orders of magnitude, while the open-circuit voltage (*V*_OC_) tripled. Building directly upon this foundational characterization, recent advances employed magnetron sputtering for BFMO fabrication and introduced strategic inequivalent barium substitution on the bismuth site [30]. This approach, leveraging the modulation of B-site ion hybridization and induced lattice distortion, achieved substantial further improvements in photovoltaic performance metrics.

Despite those progresses, the photovoltaic parameters of ferroelectric perovskite films generally remain at the due to inherent wide band gaps, insulating properties, and defect/interface limitations. In this context, rational modulation of lattice strain and cation valence states offers a promising pathway to performance optimization. Herein, we report a strategy that introduces multivalent Pb into BFMO to couple chemical strain with ionic valence regulation. This approach produces Pb:BFMO (BPFMO) system with significantly enhanced *J*_SC_ and *V*_OC_. Structural evolution was systematically analyzed through X-ray diffraction (XRD), synchrotron radiation-based reciprocal space mapping (RSMs), X-ray reflectivity (XRR), and high-resolution high-angle annular dark-field scanning transmission electron microscopy (HAADF-STEM). Valence state and orbital hybridization changes were probed by X-ray absorption spectroscopy (XAS) and X-ray photoelectron spectroscopy (XPS). Additionally, ferroelectric properties, photovoltaic characteristics, and ferroelectric photovoltaic coupling behavior are quantitatively assessed. Furthermore, the introduction of Pb^2+^ is associated with modifications to oxygen vacancy concentration in the film, the generation of Mn^3+^ ions, coordination of c-axis lattice contraction, reduction of the optical band gap, and alterations to electronic transport properties. This strategy is demonstrated to be transferable to other ferroelectric material systems, thereby establishing a novel paradigm for performance optimization in ferroelectric photovoltaics.

## Results and Discussion

**Fig. 1:**
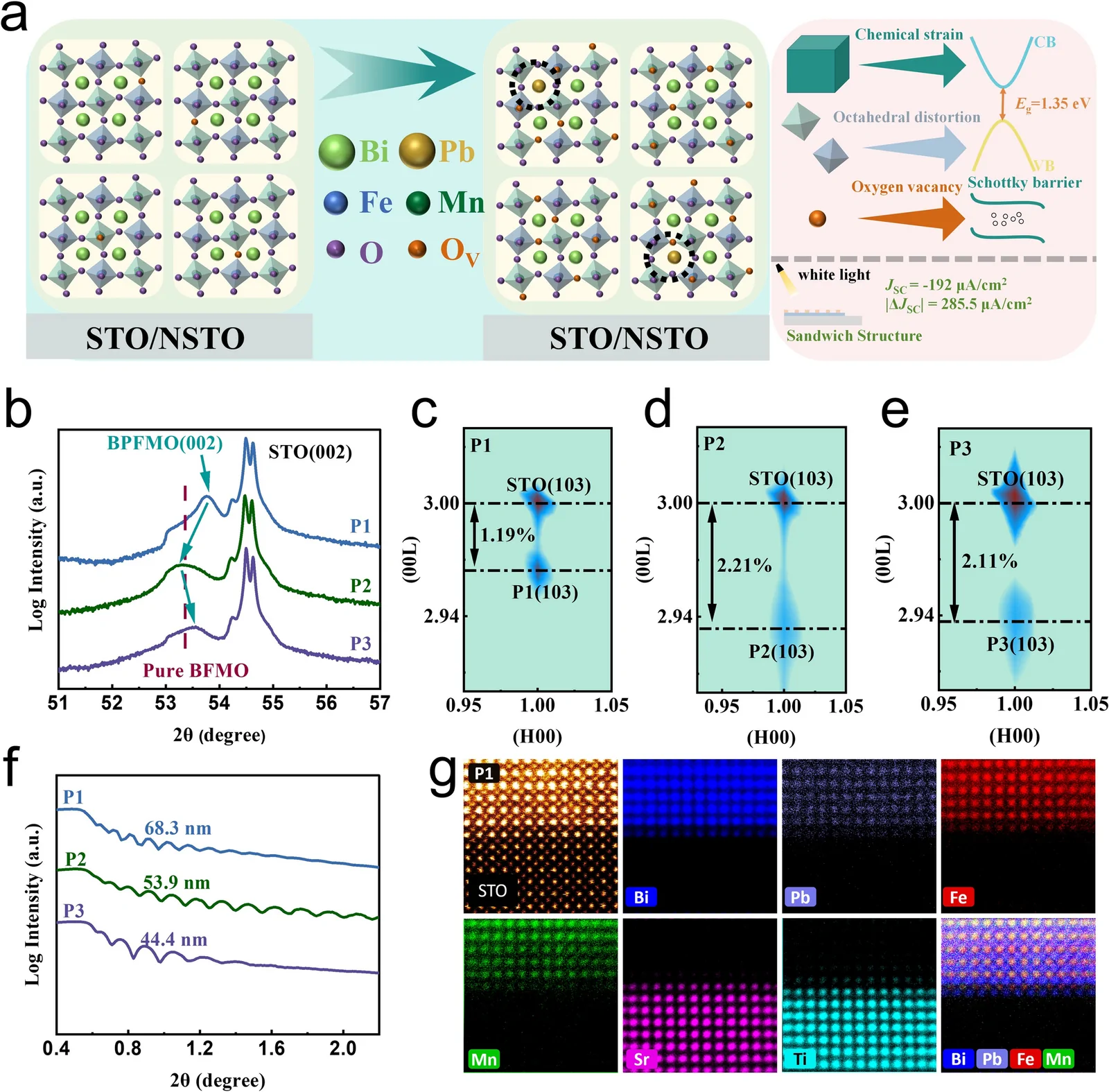
Crystal structure characterization of BPFMO double perovskite thin films. **a** Schematic diagram of possible effects after the introduction of Pb atoms. **b** Partial enlarged drawing of OOP XRD diffraction patterns of P1, P2, and P3 thin film samples on STO (001) substrate, where the magenta dashed line indicates the (002) diffraction peak position of the pure BFMO phase, and the blue arrows mark the changes of the (002) diffraction peaks of the series of samples. **c-e** Synchrotron-based RSMs along the STO substrate (103) plane for P1, P2, and P3 thin film. **f** X-ray reflectivity comparison diagram for studying the film thickness. **g** HAADF-STEM image of P1 sample and EDS map of elements in the P1 thin film and substrate, in addition, including the mapping images of Bi, Pb, Fe, and Mn elements

Employing an inequivalent substitution strategy, Pb-substituted BFMO thin films were designed. A schematic diagram illustrating the experimental feasibility is presented in Fig. 1a. Pb is usually introduced into perovskite materials in the form of Pb^2+^ [31–33]. Based on the principle of inequivalent substitution, the introduction of Pb^2+^ is anticipated to induce modifications in the hybridization state, while simultaneously exerting a regulatory influence on B-site elements and oxygen vacancies [26]. Unlike alkaline earth metals cation, additional lone electron pairs are possessed by Pb^2+^, enabling the formation of strong covalent bonds with O^2−^. Consequently, alterations in the bond lengths between cations and O^2−^ are induced, accompanied by modifications to the oxygen octahedral configuration. These structural changes are expected to facilitate the regulation of the band gap and the degree of band bending within BFMO materials, thereby modulating the bulk photovoltaic effect. Furthermore, the disparity in ionic radii between Pb^2+^ (1.19 Å) and Bi^3+^ (1.03 Å) is also conducive to the induction of chemical strain [34, 35]. The schematic diagram on the right side of Fig. 1a shows the relationship between the structure, band gap and performance after Pb introduction. The coordinated role of these three aspects will be discussed later. It should be noted that although the introduction of Pb is widely accepted to occur predominantly in the Pb^2+^ form, the existence of Pb^4+^ cannot be entirely excluded. This inherent complexity complicates the research process, necessitating the application of multiple characterization methods for conclusive verification. Based on the experimental design, Pb-substituted BFMO films were deposited on SrTiO_3_ (001) and NSTO (001) substrates using magnetron sputtering under identical deposition conditions; details of the deposition process are provided in the Methods section. The stoichiometric compositions of the films were Bi_1.94_Pb_0.06_FeMnO_6_, Bi_1.88_Pb_0.12_FeMnO_6_, and Bi_1.82_Pb_0.18_FeMnO_6_. To facilitate research, the compositions were designated as P1, P2, and P3, respectively, based on Pb content within the thin films. The large-angle XRD region (10°-70°) was exclusively composed of (00L) diffraction peaks from the films and substrate peaks, indicating high-quality epitaxial growth (Fig. S1) [33]. The small-angle region (51°-57°), presented in Fig. 1b, revealed out-of-plane (OOP) lattice constant variations across the P1, P2, and P3 thin films. In comparison with pure BFMO (magenta dashed line marking in Fig. 1b), a contraction of the OOP lattice constant is first induced by the introduction of Pb. Subsequently, an initial expansion followed by contraction is observed in the OOP lattice constant as the Pb content is increased. It is noteworthy that a larger OOP lattice constant than that of pure BFMO is exhibited by sample P2. In double perovskite materials, alterations in lattice parameters are recognized as a complex process, primarily attributed to the concomitant contributions of lattice distortion, chemical strain, and charge change [26]. In addition, the introduction of the variable element Pb is recognized as introducing a distinct degree of freedom for lattice parameter modulation, mediated through alterations in Pb's intrinsic oxidation state.

RSM, utilizing synchrotron radiation, was performed on the series of samples to comprehensively analyze variations in the epitaxial state of thin films and lattice parameter variation patterns, as shown in Fig. 1c-e. The RSM patterns demonstrated that the thin films maintained a well-defined epitaxial relationship with the substrate, while the in-plane (IP) lattice constant exhibited close agreement with the substrate value (3.905 Å). Consistent tetragonal symmetry across the P1, P2, and P3 films relative to the substrate was additionally confirmed by RSM [36]. The OOP lattice constants for P1, P2, and P3 thin films were determined to be 3.959, 3.991, and 3.975 Å, respectively, showing strong consistency with corresponding XRD measurements. A minimal variation was observed in the IP lattice constants between the epitaxial films and the substrate. However, significant OOP lattice strain in the film series was demonstrated by both XRD patterns and RSM analyses. The largest OOP distortions were identified in P2 (2.21%) and P3 (2.11%), whereas that of P1 was measured at only 1.19%. This differential strain behavior is considered to originate from lattice distortions, alterations in chemical states, and chemically induced strain resulting from lead incorporation. The epitaxial growth mode of the thin films was confirmed by RSM and XRD analyses, with coherent epitaxial strain being established across all thin films. Consequently, variations in OOP lattice parameters are interpreted as quantitative indicators of chemical strain within the films. Relative to the pure BFMO (OOP lattice constant ≈ 0.3986 nm) reference, distinct positive chemical pressures were observed in the P1 and P3 systems. As quantified by Eq. (S2), chemical pressures relative to pure BFMO of + 0.886, − 0.035, and + 0.530 GPa were measured for P1, P2 and P3, respectively. A profound influence on both crystalline structure and functional properties of thin-film materials is exerted by chemical strain [37]. Detailed alterations to these systems will be discussed subsequently.

The thicknesses of the P1, P2, and P3 thin films were determined by X-ray reflectivity (XRR) measurements (Fig. 1f). Analysis of the spectra revealed respective thicknesses of 68.3, 53.9, and 44.4 nm for the three thin films. A progressive reduction in film thickness was observed with increasing lead content, a trend documented in alternative thin film deposition systems [26]. This is because the deposition parameters were optimized primarily for Bi, Fe, Mn, and O species kinetics. Consequently, elevated target Pb concentrations were correlated with reduced growth rates, resulting in diminished film thicknesses under identical deposition durations. The epitaxial structure between the P1 film and the substrate is confirmed by HAADF-STEM image presented in Fig. 1g. Successful incorporation of lead at the A-site is demonstrated by energy-dispersive X-ray analysis (EDS). Furthermore, occupancy of the A-site by Bi and Pb, and the B-site by Fe and Mn, is indicated by EDS analysis. However, due to the similar ionic radii of Fe and Mn, complete B-site ordering cannot be definitively determined at this time. The spatial distribution of oxygen, along with elemental mappings for Bi and Pb, is further provided in Fig. S2. Additionally, large-scale HAADF-STEM images of the P1 film are provided in Fig. S3, confirming both the sample thickness and the presence of a good epitaxial growth relationship. The crystal structure of the P1 thin film is verified by local fast Fourier transform (FFT) and corresponding local-scale HAADF-STEM image acquired from a microscopic perspective. Finally, geometric phase analysis (GPA) reveals the presence of localized compressive and tensile chemical strains within the P1 thin film. A foundation for subsequent research is established by these structural characteristics.

**Fig. 2:**
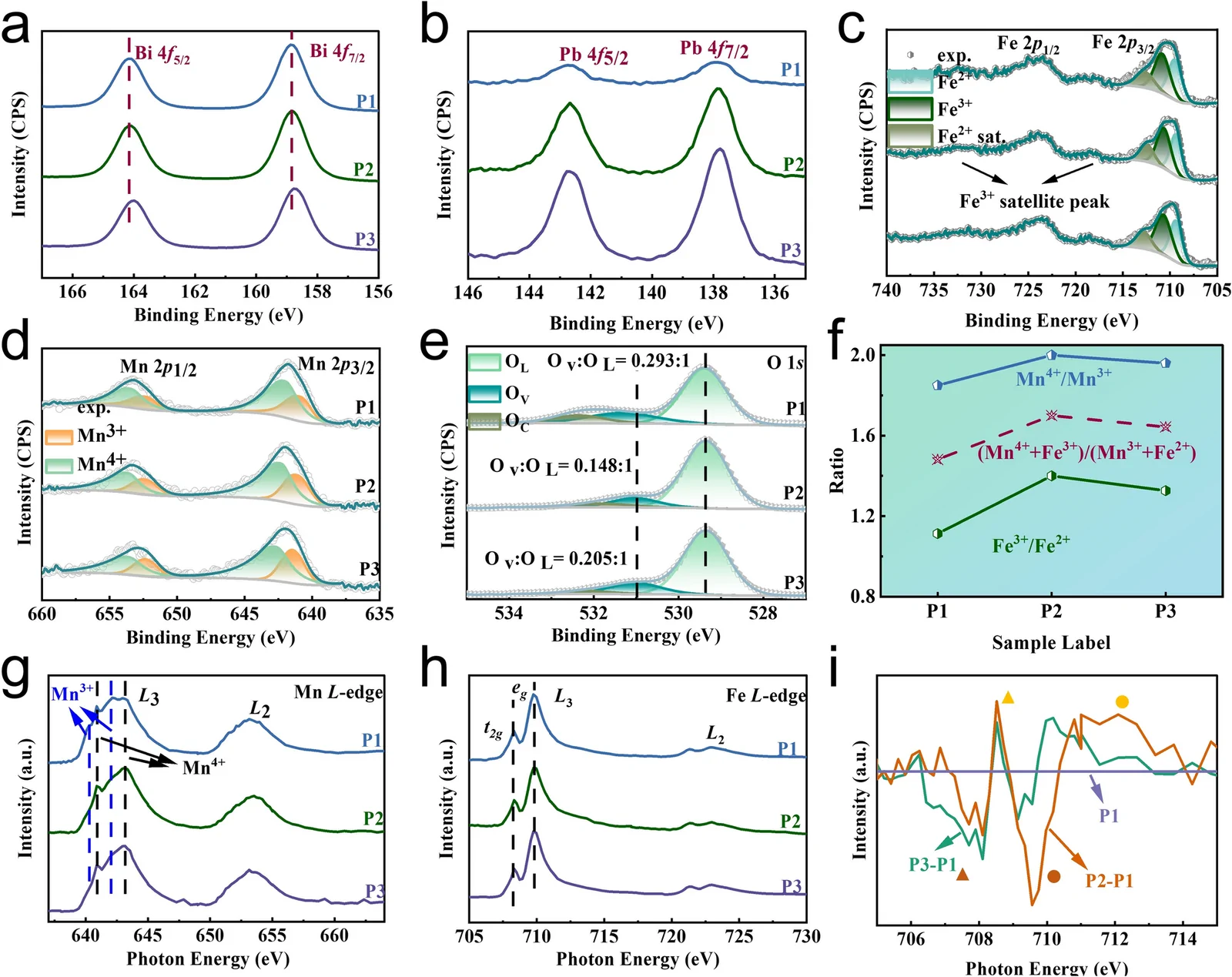
Composition and orbital hybridization of BPFMO double perovskite thin films. **a** Bi 4*f* XPS spectra of P1, P2, and P3 thin films. CPS: counts per second. **b** Pb 4*f* XPS spectra of P1, P2, and P3 thin films. **c** Fe 2*p* energy level XPS spectra of P1, P2, and P3 films and the deconvolution fitted peaks obtained from their inverse convolution are classified as Fe^2+^ peaks, Fe^2+^ satellite peaks, and Fe^3+^ peaks in the Fe 2*p*_3/2_ region. **d** Mn 2*p* energy level XPS spectra of P1, P2, and P3 films and the deconvolution fitted peaks obtained from their inverse convolution are classified as Mn^3+^ peaks and Mn^4+^ peaks in the Fe 2*p* region. **e** O 1*s* XPS spectra of P1, P2, and P3 thin films show that the curves are resolved into three peaks, corresponding to O_L_, O_V_, and O_C_. **f** Trend diagram of Fe, Mn and (Fe + Mn) elemental valence states ratios of Fe and Mn elements in the P1, P2, and P3 films. **g** Mn *L*_2,3_-edge XAS spectra of P1, P2, and P3 thin films, with red and blue dashed lines representing the positions of the characteristic peaks of Mn^4+^ and Mn^3+^, respectively. **h** Fe L_2,3_-edge XAS spectra of P1, P2, and P3 thin films. **i** Fe L_3_ edge profiles of P2 and P3 films are normalized and then subtracted from the normalized edge profiles of P1. The solid brown or yellow circles and triangles are used as markers to indicate the positions of the orbital components. According to the energy positions of the orbital components, brown and yellow represent the OP (*d*_3z_^2^_−r_^2^, *d*_yz_, and *d*_xz_) orbital component and the IP (*d*_x_^2^_−y_^2^, *d*_xy_) orbital component, respectively. Among them, the triangle and the circle, respectively, represent the *t*_2g_ and *e*_g_ energy levels of the Fe 3*d* orbitals

The chemical stoichiometry, elemental valence states, lattice oxygen evolution, and resultant structural modifications (including lattice distortion, iron ion displacement, and Fe–O octahedral hybridization changes) in Pb-substituted BFMO double perovskite films were investigated through XPS and XAS. Although XPS is regarded as semi-quantitative, variation trends between samples could be reliably assessed via semi-quantitative analysis of Bi 4*f*, Pb 4*f*, Fe 2*p*, Mn 2*p*, and O_L_ 1*s* peak areas in survey spectra. As summarized in Table S1, elemental composition was determined through these computations, confirming all samples exhibited stoichiometric ratios consistent with A^2+^B′B″O_6_ double perovskite configurations. Survey XPS spectra confirmed the presence of Bi, Pb, Fe, Mn, and O in all specimens, with progressively increasing Pb content observed across the series (Fig. S4). All core-level spectra were calibrated against the C 1*s* adventitious carbon peak (284.8 eV) to correct for binding energy shifts. The Bi 4*f* XPS spectra of P1, P2, and P3 films were characterized by two principal spin–orbit components (Bi 4*f*_5/2_ and Bi 4*f*_7/2_), confirming the predominant existence of bismuth as Bi^3+^ cations (Fig. 2a). As emphasized by the dotted guidelines in Fig. 2a, the Bi 4*f*_7/2_ binding energy of the P3 thin film (158.68 eV) was shifted toward lower energies relative to P1 and P2 counterparts (158.88 eV). Such spectral shifts are recognized in XPS analysis as indicators of altered charge density surrounding atomic nuclei, implying modifications in chemical bonding environments. Given Bi^3+^ as its most stable oxidation state, the observed chemical state perturbation of Bi in P3 is attributed primarily to defects (such as a few Bi vacancies) formation. This indicates that the content of Pb should not continue to increase. The Pb 4*f* XPS spectra of P1, P2, and P3 films are presented in Fig. 2b. A progressive enhancement in Pb 4*f* peak intensity was observed with increasing Pb content, correlating with compositional variation. For enhanced clarity regarding these spectral shifts, comparative peak position analysis is provided in Fig. S4b. As shown in Fig. S2, a small but discernible shift of the peak positions toward lower binding energies (ΔE ≈ 0.35 eV) is observed in the P2 and P3 samples compared to the P1 sample. It is recognized that Pb^2+^ exhibits a higher binding energy than Pb^4+^ [38]. Consequently, this subtle shift may be interpreted as indicative of the presence of Pb^4+^ ions in the P2 and P3 samples, which is consistent with the anticipated progression. However, due to the extremely weak magnitude of the shift and the inherent proximity of the characteristic binding energies for Pb^2+^ and Pb^4+^, spectral deconvolution is often prone to overfitting, yielding unreliable speciation results. Therefore, analysis was restricted to a qualitative interpretation based solely on the observed peak position changes. This energy displacement can be interpreted as evidence of Pb oxidation state modulation, indicating that lead incorporation may achieve charge balance through valence state adaptation within the double perovskite lattice.

The deconvoluted Fe 2*p* and Mn 2*p* XPS spectra of P1, P2, and P3 films are presented in Fig. 2c, d, respectively. For the Fe 2*p*_3/2_ region, three constituent peaks were resolved to Fe^2+^ satellite peak, Fe^3+^ and Fe^2+^ across all samples. Similarly, the Mn 2*p*_3/2_ spectra were fitted with two peaks for Mn^4+^ and Mn^3+^. The coexistence of multiple transition metal oxidation states (Fe^3+^/ Fe^2+^ and Mn^4+^/ Mn^3+^) was confirmed within the double perovskite thin films. For qualitative comparison of transition metal valence evolution across the film series, deconvoluted elemental oxidation state ratios are plotted in Fig. 2f, with full numerical data provided in Tables S1 and S2. Following the introduction of Pb, the concentrations of Fe^3+^ and Mn^4+^ were both observed to exhibit a nonlinear trend, characterized by an initial increase followed by a subsequent decrease. The valence state variations of the B-site elements were found to be consistent with the changes in oxygen vacancy concentration. Consequently, the introduction of Pb was demonstrated to induce lattice distortion and generate oxygen vacancies, thereby stabilizing the B-site elemental valence states. Complementary analysis of oxygen speciation (Fig. 2e) revealed that the O 1*s* spectrum could be deconvoluted into three components: lattice oxygen (O_L_), oxygen vacancies (O_V_), and surface-adsorbed species (O_C_). The O_V_/O_L_ ratios were quantified as 0.293 (P1), 0.148 (P2), and 0.205 (P3); full numerical data are provided in Table S1 and Table S2. Following the introduction of lead (Pb), a nonlinear variation in oxygen vacancy concentration is observed. The maximum value is attained in composition P1, while the minimum manifests in P2. This behavior aligns with alterations in the oxidation state of B-site elements, thereby effectively preserving the oxidation state equilibrium. In double perovskite oxides, valence state variations and functional modifications of B-site cations and oxygen anions are fundamentally interconnected. The valence state alterations induced by lead (Pb) introduction are observed to exert profound influences on the material's electronic structure, ultimately dictating its functional performance.

In BPFMO double perovskite films, Fe and Mn elements are accommodated at the B-sites. Consequently, modifications in orbital hybridization are reflected in structural distortions of Fe-O_6_ and Mn-O_6_ octahedra, leading to alterations in both crystalline symmetry and elemental oxidation states. Such coordination changes may further perturb the electronic band structure. Therefore, XAS was employed to probe transitions from core levels to unoccupied states via photon energy modulation. The Mn L-edge XAS spectra of P1, P2, and P3 films are presented in Fig. 2g, where spin–orbit coupling (Δ≈ 11.8 eV) manifests as distinct *L*₃ (640–645 eV) and *L*₂ (650–655 eV) absorption edges. The multiplet splitting within the Mn–*L*₃ region is diagnostic of mixed-valence states (Mn^3+^/Mn^4+^), with characteristic spectral features peak at 640.30 eV (Mn^3+^ shoulder peak), 642.03 eV (Mn^3+^ main peak), 640.93 eV (Mn^4+^ shoulder peak), and 643.18 eV (Mn^4+^ main peak). The red line and blue line in Fig. 2g mark the characteristic peaks of Mn^4+^ and Mn^3+^, respectively. In addition, XAS shows that in the P1, P2, and P3 thin films, the main peak of Mn^3+^ (642.03 eV) and the shoulder peak (640.30 eV) in the P1 sample are the most obvious, while the main peak of Mn^4+^ (643.18 eV) and the shoulder peak (640.93 eV) in the P2 sample are the most obvious, indicating that the content of Mn^3+^ is the highest in the P1 sample, and the content of Mn^4+^ is the highest in the P2 sample. This result is also confirmed by XPS.

The Fe *L*-edge XAS spectra of the P1, P2, and P3 films are presented in Fig. 2h, delineated into the *L*_3_-edge and L₂-edge regions. Within each region, distinct *e*_g_ and *t*_2g_ peaks are observed, indicating that the Fe element within the films predominantly exists in the Fe^3+^ state. However, the existence of Fe^2+^ ions is also inevitably present, owing to the existence of oxygen vacancies and other structural defects. To comprehensively elucidate changes in the orbital hybridization of the Fe–O octahedra across the P1, P2, and P3 films, the Fe *L*_3_-edges were normalized using the e_g_ peak. Subsequently, the differential spectra between the normalized Fe *L*_3_-edge XAS intensities of the P2 and P3 films and that of the P1 film are presented in Fig. 2i. The differential spectra of P3 relative to P2 are presented in Fig. S5. Through these spectra, the process peak splitting of orbitals within the Fe–O octahedra is revealed. Consistent with crystal field theory, the Fe-3*d* energy level in BFO-based films is subdivided into an OOP orbital component (*d*_3z_^2^_−r_^2^, *d*_yz_, *d*_xz_) and an IP orbital component (*d*_x_^2^_−y_^2^, *d*_xy_), where *d*_xy_ and *d*_xz_ exhibit degenerate orbital energies. Prior studies have established that relative to pure BFO, the OOP orbital energy of BFMO films is significantly elevated, while the IP orbital energy is reduced, corresponding to OOP compression of the Fe–O octahedron [26]. As shown in Fig. 2i, both *e*_g_ and *t*_2g_ orbital energies in P2 and P3 films exhibit enhanced splitting compared to the P1 reference. This enhancement corresponds to a lengthened OOP orbital bond length (reducing OOP energy) and a shortened IP orbital bond length (increasing IP energy). Typically, enhanced splitting within the Fe-3*d* manifold is associated with intensified OOP elongation or tilt distortion of the Fe–O octahedron. The incorporation of Pb into the perovskite lattice is associated with two primary mechanistic effects. Firstly, the coordinated changes in the valence states of B-site element ions and O ions can be regulated through the introduction of Pb, thereby enabling effective control over the mixed valence states of the B-site elements. However, since changes in the valence state of the B-site induce octahedral distortion and lattice distortion, and since the introduction of elements possessing differing ionic radius (such as Pb) also causes lattice distortion, the average valence state of Pb may be slightly elevated upon its intensive introduction. Consequently, the introduction of excessive amounts of Pb is not considered appropriate. This divergence suggests that the orbital splitting energy in P3 may be amplified relative to P2, potentially arising from modified electronegativity or symmetry of Fe ions in P3 (Fig. S7).

**Fig. 3:**
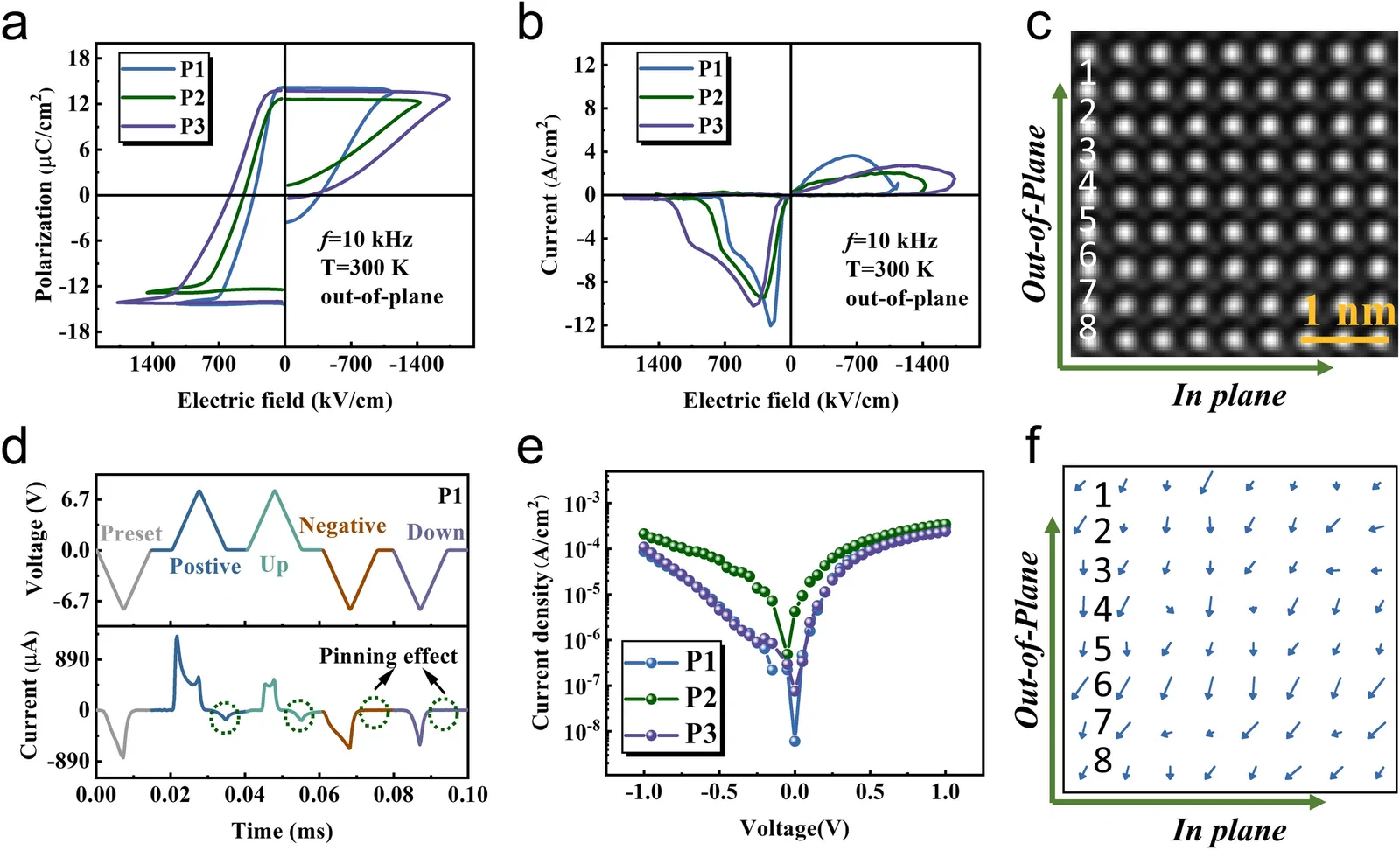
Electrical properties of BPFMO double perovskite thin films. **a** P-E loops on the bottom electrode of NSTO for P1, P2, and P3 thin films in PUND mode at room temperature and 10 kHz. **b** Switching current loops of P1, P2, and P3 thin films in PUND mode at room temperature and 10 kHz. **c** HAADF-STEM image with large distortion characteristics for BPFMO double perovskite thin films. **d** Oscillogram of polarization current (bottom) and triangle wave voltage range from − 8 to + 8 V; top) measured in PUND mode with time. **e** Leakage current curves of P1, P2, and P3 thin films. **f** Polarization vector analysis of the B-site ionic positions for the HAADF-STEM image in **c**

The OOP ferroelectric properties of P1, P2, and P3 films were characterized using Pt/BPFMO/NSTO heterostructures. Polarization measurements were conducted via the Positive-Up-Negative-Down (PUND) method, which was employed to mitigate contributions from non-ferroelectric artifacts to the hysteresis loop, an advantage over conventional polarization–electric field (P-E) loop characterization [39]. As shown in Fig. 3a, under ambient conditions at 10 kHz with leakage current compensation, double remanent polarization (2P_r_) values of 23.05, 20.99, and 18.01 μC cm^−2^ were recorded for P1, P2, and P3 films, respectively. Significant asymmetry in the hysteresis loops under positive and negative biases was observed, attributable to electrode work function mismatch between the Pt top electrode and NSTO bottom electrode [40, 41]. This interfacial asymmetry induces dissimilar band bending at each electrode-film junction, generating a Schottky barrier that establishes an inherent electric field gradient across the device architecture. Under the influence of the built-in electric field and the asymmetric electron injection pinning of ferroelectric domains effect, polarization switching completely (positive orientation) and polarization suppression (negative orientation) are induced in ferroelectric domains along opposing directions. Consequently, under experimental conditions, complete domain reversal in the negative polarization direction is impeded. During ferroelectric polarization, charge migration and redistribution occur within the material, generating switching current signals that serve as direct evidence of polarization dynamics. The switching current evolution for P1, P2, and P3 films is presented in Fig. 3b. The consistency between switching current profiles and hysteresis loops verifies the reliability of the measured polarization values. Analogous to the hysteresis asymmetry, switching current curves also exhibit bias-direction asymmetry due to the interfacial barrier. Owing to distortion of the hysteresis loop in the negative field regime, comparative analysis of coercive fields (*E*_C_) is confined to the positive polarization region. The + *E*_C_ values for P1, P2, and P3 films are quantified as 336.8, 431.3, and 585.9 kV cm^−1^, respectively, demonstrating a monotonic increase with Pb content. The coercive electric field in thin-film ferroelectric materials is strongly influenced by thickness dependence. It is generally observed that the coercive electric field of ferroelectric films increases as the film thickness is reduced [42]. Therefore, the change in coercive electric field following Pb introduction is attributed not only to the synergistic effects of oxygen vacancies and chemical strain, but is also modulated by the film thickness (Fig. 1f). The PUND triangular wave test waveform (top panel) and synchronous current evolution (bottom panel) are presented in Fig. 3c. Leakage current characteristics for P1, P2, and P3 films are shown in Fig. 3d, where interfacial asymmetry originating from dissimilar top (Pt) and bottom (NSTO) electrodes is observed. Excellent insulating behavior is demonstrated across all films, though significantly elevated leakage is noted in the P2 specimen. According to XPS spectral analysis, this anomalous leakage in P2 exhibits the lowest oxygen vacancy concentration. Therefore, the leakage current of P2 is attributed not to oxygen vacancy concentration but to modified charge transport mechanisms. For quantitative assessment of insulation properties, near-zero-field current–voltage (*J*-*V*) curves were linearly fitted (Fig. S6). Resistivities of ∼10^10^ Ω cm were determined for P1 and P3 films, exceeding the 10^9^ Ω cm value measured for P2. In addition, the atomic-scale polarization configuration was obtained through analysis of B-site atomic polarization vectors in HAADF-STEM images, utilizing an independently developed MATLAB script. As the script’s calculations are dependent on HAADF-STEM contrast, regions exhibiting structural distortions along the OOP direction (Fig. 3c) were randomly selected following substrate compliance with script requirements. The resulting polarization vectors are presented in Fig. 3f, where arrows denote B-site atomic polarizations. Significant ion displacement of B-site atoms within the analyzed region was observed. The polarization vectors exhibit a distinct net polarization in the OOP direction, accompanied by nonzero polarization projections in the IP direction. Ferroelectric polarization in BFO is primarily attributed to the lone electron pairs of Bi^3+^ ions and the relative displacement of the Fe–O octahedra along the < 111 > direction [43]. Similarly, the origin of ferroelectricity in BFMO is considered analogous. Correlation with strain levels, as indicated by the GPA spectra in Fig. S3, further supports these findings [44].

To elucidate conduction mechanisms in Pt/BPFMO/NSTO heterostructures, current density–electric field (*J*-*E*) characteristics were modeled using space-charge-limited conduction (SCLC) theory [45, 46]. The ln|*J*| versus ln|*E*| relationships under positive bias are displayed in Fig. S8a. Identical conduction mechanisms were observed across P1, P2, and P3 thin films. Below 300 kV cm^−1^, ohmic behavior was exhibited. Under negative bias (Fig. S8b), interfacial conduction at film/NSTO boundaries was probed. Progressive field intensification triggered sequential transitions: ohmic-to-SCLC followed by trap-filled-limit behavior was demonstrated in P1 and P3 films. Conversely, ohmic conduction was entirely absent in P2. This deviation indicates that compositional modification and resultant chemical strain in P2 fundamentally reconfigure charge transport pathways while simultaneously altering interfacial electrostatics at the P2/NSTO heterojunction. In conclusion, the formation of an interfacial electric field has been unequivocally confirmed through a systematic analysis of hysteresis loop asymmetry, switching current dynamics, and leakage behavior.

**Fig. 4:**
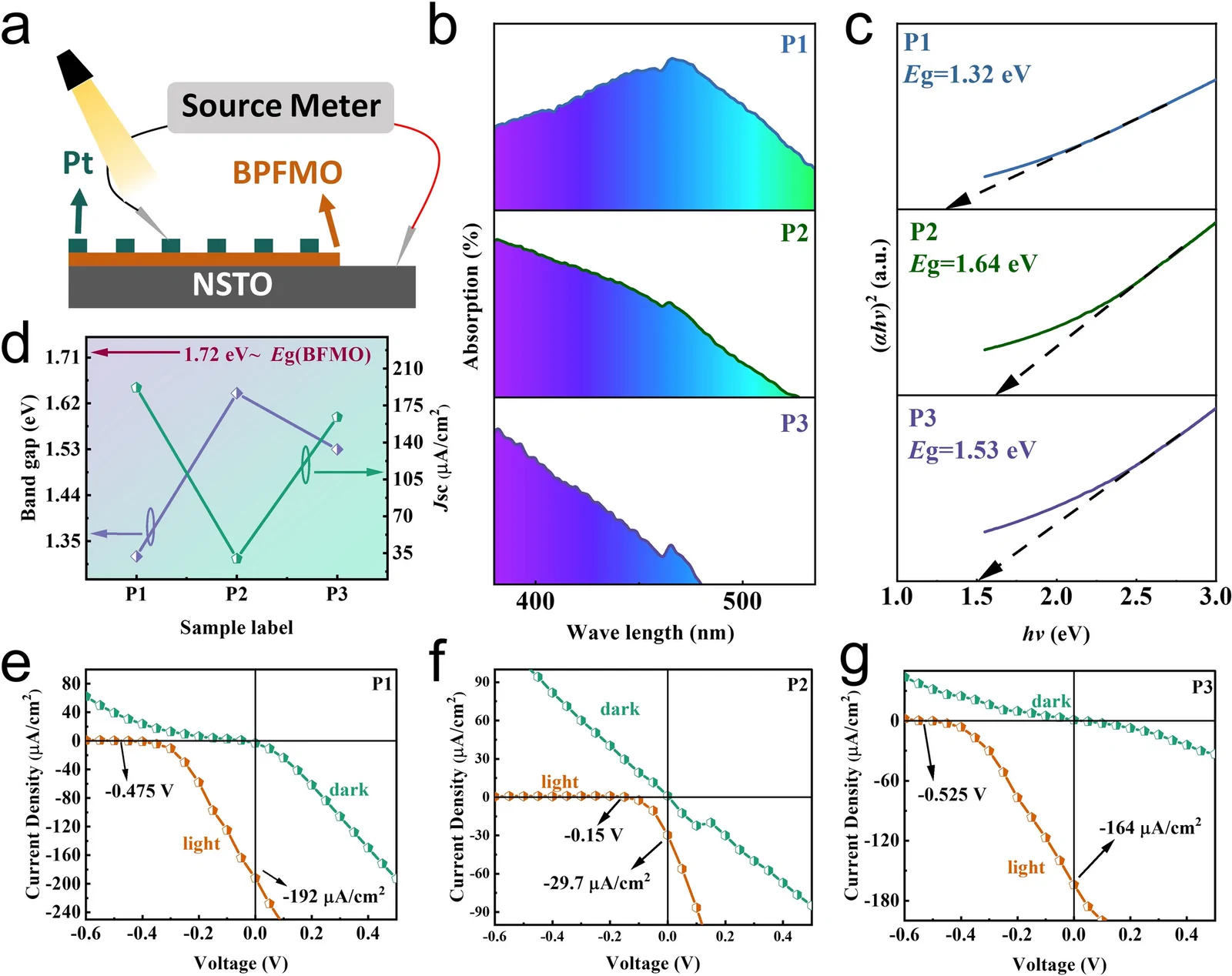
Photovoltaic properties of BPFMO double perovskite thin films. **a** Schematic diagram of Pt/BPFMO/NSTO device layout structure. **b** Absorption coefficient (*α*) versus wavelength curve of P1, P2 and P3 double perovskite thin films. **c** (*αhν*)^2^ versus photon energy curves of P1, P2 and P3 double perovskite thin films. **d** Statistical diagrams of the trends of the *E*_g_ values (the gray arrow indicates the band gap of pure BFMO) and the *J*_SC_ of P1, P2, and P3 thin films. *J*-*V* curves of **e** P1 thin film, **f** P2 thin film, **g** P3 thin film under dark and the light intensity of 80 mW cm^−2^

The schematic diagram of the Pt/BPFMO/NSTO device employed in photovoltaic and electrical performance characterization is presented in Fig. 4a. The optical absorption characteristics of P1, P2, and P3 films were evaluated through absorbance versus photon energy functional analysis (Fig. 4b). Potential underestimation of bandgap values is attributed to attenuated absorption peaks caused by high reflectivity of single-crystal substrates. UV–visible absorption spectra were fitted employing a Tauc–Lorentzian model (Fig. 4c), wherein direct bandgaps were derived from linear extrapolation of (*αhν*)^2^ versus hν plots to the energy axis intercept [47]. As demonstrated in Fig. 4c, the direct bandgap of BPFMO films is modulated by Pb content variation, exhibiting an initial increase followed by reduction. The minimal bandgap of 1.32 eV was recorded for P1, whereas P2 and P3 films were characterized by bandgaps of 1.64 and 1.53 eV, respectively. Moreover, in contrast to pure BFMO films investigated previously (1.72 eV), reductions in the direct band gaps of lead-incorporated P1, P2, and P3 films were observed. Within perovskite materials research, these band gap is regarded as comparatively low (< 1.5 eV) [10]. From a crystallographic perspective, lattice contraction is induced by positive chemical strain in P1 and P3 relative to P2. Positive chemical strain is demonstrated to enhance optical absorption capacity while simultaneously mediating bandgap reduction through lattice distortion mechanisms, and the relative positions of the valence band maximum (VBM) and conduction band minimum (CBM) are adjusted through lattice contraction in this process [48]. This phenomenon can also be explained by considering bond-length shortening and changes in orbital hybridization, wherein enhanced Mn^3+^-O^2−^ covalency is induced under compressive strain states. Mechanistically, bond lengths and orbital hybridization states are reconfigured by Pb incorporation, while bandgap narrowing is concurrently mediated through enhanced electron delocalization resulting from increased Mn^3+^ ion concentration. The interdependence of direct bandgap (*E*_g_) and *J*_SC_ for P1-P3 films is presented in Fig. 4d. A pronounced inverse correlation is observed, wherein reduced *E*_g_ is associated with enhanced *J*_SC_. This phenomenon is attributed to extended photon harvesting within the visible spectrum (400–700 nm), facilitating increased charge carrier generation. *J*-*V* characteristics measured at 80 mW cm^−2^ are summarized in Fig. 4e-g, revealing *J*_SC_ values of − 192, − 29.7, and − 164 μA cm^−2^ with corresponding *V*_OC_ of 0.475, 0.15, and 0.525 V for P1, P2, and P3, respectively. Previous studies have suggested that the ferroelectric photovoltaic effect in thin film materials is related to thickness [49–51]. This phenomenon can be attributed to alterations in the electronic transmission mechanism, changes in the total volume of illuminated thin films, and competitive effects resulting from photonic reception at the heterojunction interface (specifically the BPFMO/NSTO interface, as previously defined in this study). However, substantial thickness differences (typically exceeding 100 nm) were investigated in prior studies, wherein a consistent pattern of enhanced photovoltaic response was observed in thinner films. In contrast, the thickness variations examined in the present study were confined to a narrow range (44.4–68.3 nm). Within this range, a significant non-monotonic dependence of the photocurrent response on thickness was observed, which is different from the usual situation where photovoltaic performance monotonically increases with decreasing thickness. Consequently, it is concluded that the influence of film thickness on the photovoltaic behavior is of secondary importance under the conditions investigated. When compared with pure BFMO (synthesized under identical preparation conditions and measured at 100 mW cm^−2^ illumination intensity, exhibiting a *J*_SC_ of 3.61 μA cm^−2^ and a *V*_OC_ of 0.16 V), the *J*_SC_ and *V*_OC_ of the BPFMO film were enhanced to varying degrees. Specifically, the *J*_SC_ of the P1 film was observed to be more than 53 times higher than that of pure BFMO, while the *V*_OC_ was measured to be more than three times that of pure BFMO. These improvements indicate that the introduction of Pb elements was observed to exert a significant influence on the photovoltaic performance of the BFMO material system. Furthermore, as the Pb content was increased, both *J*_SC_ and *V*_OC_ of BPFMO thin films exhibited an initial decline followed by an enhancement. This non-monotonic trend is attributed to variations in light absorption capacity associated with differing Pb concentrations. Light-switching chronoamperometry profiles (Fig. S9) further validate photo-response stability.

**Fig. 5:**
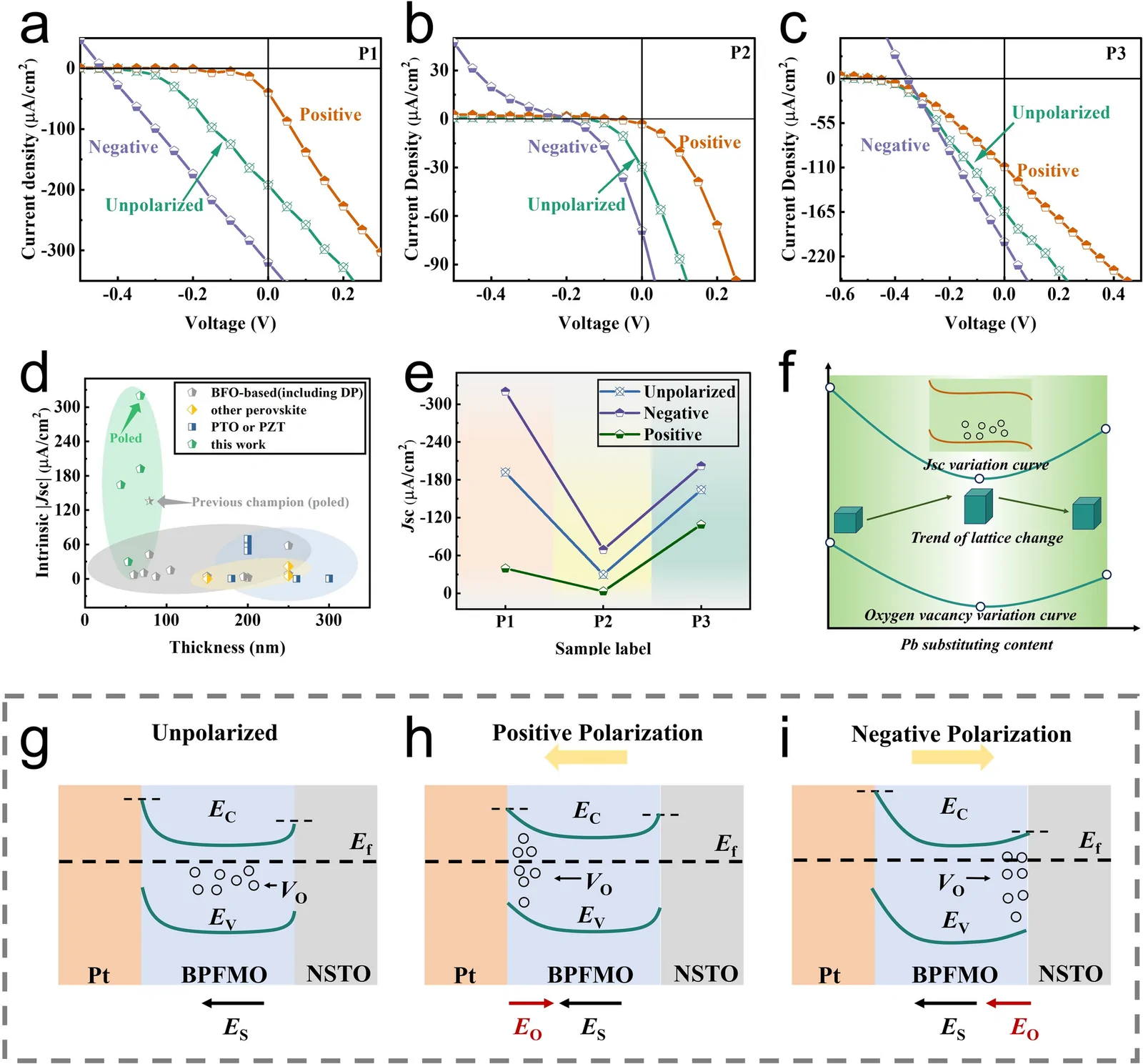
Ferroelectric photovoltaic properties of BPFMO double perovskite thin films. *J*-*V* curves of **a** P1 thin film, **b** P2 thin film, and **c** P3 thin film under unpolarized, positive polarization, and negative polarization state under illumination with 80 mW cm^−2^. **d** Comparison of *J*_SC_ and thickness of ferroelectric photovoltaic devices based on the reported [26–28, 52–56]. **e** Statistical plot of unpolarized *J*_SC_, positive polarization *J*_SC_, and negative polarization *J*_SC_. **f** A schematic diagram showing the changes in oxygen vacancies, the changes in the OOP lattice constant, and the variation in *J*_SC_. Schematics of** g** energy band diagrams across the Pt/BPFMO/NSTO device in the unpolarized state,** h** the positive polarization state, and **i** the negative polarization state. *E*_V_, *E*_C_, *E*_f_, *E*_S_, and *E*_O_ represent the valence band, the conduction band, the Femi level, the start Schottky barrier-induced field, and the built-in field formed by oxygen vacancies, respectively

The ferroelectric polarization dependence of photovoltaic properties was systematically investigated through *J*-*V* characterization of P1-P3 thin films devices under three distinct poling states (unpoled, positive polarization, negative polarization) at 80 mW cm^−2^ illumination (Fig. 5a-c). Significant deviations from intrinsic characteristics were observed following 60 s polarization pulses (the polarization voltage is ± 9 V), with pronounced modulations in the *J*_SC_. Under negative poling, the *J*_SC_ enhancement was recorded, elevating from intrinsic values of − 192, − 29.7, and – 164 μA cm^−2^ to -320, -69.1, and − 202 μA cm^−2^ for P1, P2, and P3, respectively. Conversely, positive poling suppressed *J*_SC_ to − 39.5, − 3.04, and − 109 μA cm^−2^. This bidirectional tunability demonstrates an electric field-modulated photovoltaic effect in Pt/BPFMO/NSTO heterostructures, wherein the *J*_SC_ is controllably regulated by polarization orientation. In Fig. 5d, the numerical relationship is presented between the thickness (within 300 nm), the composition of single-layer perovskite films, and the resultant photovoltaic performance under 100 mW cm^−2^ illumination. (The illumination in this work is 80 mW cm^−2^.) The initial *J*_SC_ performance was significantly enhanced by the P1 film, surpassing that exhibited by previously reported perovskite-based ferroelectric photovoltaic devices with single absorber layer. The significant ferroelectric photovoltaic response in the BPFMO thin film at lower thicknesses (≤ 100 nm), indicating significant implications for practical application of small-sized devices. Compared to conventional BFO-based ferroelectric photovoltaic devices, the substantially enhanced *J*_SC_ of the BPFMO thin film is expected to promote the further development of ferroelectric photovoltaic technology. To facilitate comparison of the photovoltaic and ferroelectric photovoltaic responses, the *J*_SC_ values for the P1, P2, and P3 thin films are provided in Fig. 5e for the un-polarized, positively polarized, and negatively polarized states. These results demonstrate that, in addition to possessing a large initial photovoltaic response, the amplitude of the electric-field-modulated current density in sample P1 (quantified as |Δ*J*_SC_| =|*J*_UP_—*J*_DOWN_|) was also found to be large. To advance the understanding of ferroelectric photovoltaics, the variation laws of oxygen vacancy concentration, *J*_SC_, and the OOP lattice constant are constructed in Fig. 5f. Furthermore, schematic diagrams illustrating band structure modulation driven by oxygen vacancy migration are presented in Fig. 5g-i. The structural model was constructed based on uniformly distributed oxygen vacancies within BPFMO films (Fig. 5g). Schottky barriers were formed at electrode interfaces due to work function disparity between NSTO (*φ* ≈3.6 eV) and Pt (*φ* ≈ 5.6 eV) [57, 58], generating an intrinsic built-in field (*E*_S_) across the film. Upon positive poling (Fig. 5h), oxygen vacancies (positively charged defects) were driven to redistribute along the polarization vector, accumulating at the Pt/BPFMO interface. This process was established a *V*_O_-enriched barrier field (*E*_O_) anti-parallel to *E*_S_. The counter-aligned *E*_O_ thereby reduced the effective barrier height, inhibiting photogenerated carrier separation. Consequently, a reduction in the *J*_SC_ of the BPFMO device was accompanied by suppression of the diode rectification effect. Following negative polarization (Fig. 5i), oxygen vacancies were driven toward the BPFMO/NSTO interface, resulting in cooperative alignment of the *V*_O_-induced field (*E*_O_) and intrinsic Schottky field (E_S_) [59]. Field superposition was achieved, elevating the effective barrier height. Consequently, the synergistic interplay between the defect-induced built-in field (*E*_O_) and the electrode asymmetry-derived interfacial field (*E*_S_) enables electric field-tunable *J*-*V* characteristics. Meanwhile, the photovoltaic response modulated by the electric field is also influenced by the concentration of oxygen vacancies. A stronger ability to modulate the potential barrier following migration is exhibited by higher concentrations of oxygen vacancies. Consequently, a larger modulation amplitude of the |Δ*J*_SC_| is observed in the P1 film.

## Conclusion

In conclusion, epitaxial BPFMO thin films were fabricated on STO (001) substrates via magnetron sputtering. It has been demonstrated that systematic variation of the Pb content enables modulation of the crystal structure and ionic valence states within the films. These compositional alterations are found to induce significant changes in chemical strain and orbital hybridization characteristics, consequently allowing precise control over functional properties, specifically ferroelectricity and photovoltaic performance. The photoelectric properties observed in this study are primarily attributed to two factors. Firstly, the introduction of Pb induces chemical tensile strain, leading to an effective reduction in the OOP lattice constant. Secondly, a decrease in the valence state at the B-site, specifically an increased concentration of Mn^3+^ ions, is observed. At reduced OOP lattice constants and lower valence states, the electronic delocalization properties are enhanced, resulting in effective band gap narrowing and an improved ferroelectric photovoltaic response. Consequently, the coordinated modulation of chemical strain and ionic valence state is demonstrated to be a universal strategy for controlling the ferroelectric and ferroelectric photovoltaic properties of thin-film materials. This approach significantly enhances the application potential of ferroelectric materials, particularly within the field of ferroelectric photovoltaics.

## Supplementary Information

Below is the link to the electronic supplementary material.

Supplementary file1 (DOCX 4602 KB)
