# Supplementary material for: Chemo-Strain Valence Engineering for Boosting Photovoltaic Response in Double Perovskite Epitaxial Films
Source: Nanomicro Lett. 2026 Mar 4;18:271. doi: 10.1007/s40820-026-02105-y (PMC12961004; doi:10.1007/s40820-026-02105-y)
Supplement: Supplementary file 1 — Supplementary file1 (DOCX 4602 KB) [file 40820_2026_2105_MOESM1_ESM.docx]

Supporting Information for

**Chemo-Strain Valence Engineering for Boosting Photovoltaic Response in Double-Perovskite Epitaxial Films**

Yonghui Wu^1+^, Jie Tu^1+^, Jing Xia^2^, Xudong Liu^1,3^, Longyuan Shi^1^, Hangren Li^1^, Menglin Li^1^, Peng Chen^4^, Qianqian Yang^1^, Siyuan Du^1^, Pengfei Song^1^, Haiying Li^1^, Qian Zhan^4^, Xiaolong Li^5^, Jianjun Tian^1^, and Linxing Zhang^1,6*^

^1^ Institute for Advanced Materials Technology, University of Science and Technology Beijing, Beijing 100083, P. R. China

^2^ Key Laboratory of Photochemical Conversion and Optoelectronic Materials, Technical Institute of Physics and Chemistry Chinese Academy of Sciences, Beijing 100190, P. R. China

^3^ School of Electronic Science and Engineering, Collaborative Innovation Center of Advanced Microstructures, Nanjing University, Nanjing 210093, P. R. China

^4^ School of Materials Science and Engineering, University of Science and Technology Beijing, Beijing 100083, P. R. China

^5^ Shanghai Synchrotron Radiation Facility, Shanghai Advanced Research Institute, Chinese Academy of Sciences, Shanghai 201204, P. R. China

^6^ Institute of Solid State Chemistry, University of Science and Technology Beijing, Beijing 100083, P. R. China

^+^ Yonghui Wu and Jie Tu contributed equally to this work.

* Corresponding author. E-mail: [linxingzhang@ustb.edu.cn](mailto:linxingzhang@ustb.edu.cn) (Linxing Zhang)

**S1 Methods**

*Thin-film synthesis*: Bi_(2-2_*_x_*_)_Pb_2_*_x_*FMO_6_ (Pb = 0.1, 0.2, 0.5) ceramic targets were synthesized via conventional solid-state reaction at 850°C for 3 hours. To mitigate bismuth volatilization induced by the high-temperature crystallization of the perovskite phase, ~10% excess Bi_2_O_3_ was incorporated into the targets. All targets were pre-sputtered for 30 minutes to remove surface contaminants. Subsequently, BPFMO double perovskite thin films were deposited on (001)-oriented STO and NSTO substrates by radio-frequency magnetron sputtering. Deposition was performed at 650°C under 0.4 Pa pressure with an Ar/O_2_ flow ratio of 3:7 (total flow rate: 10 sccm), using a sputtering power of 90 W for 1 hour. Finally, all samples were maintained in situ for 30 minutes within a 120-Pa oxygen atmosphere.

*Structural characterizations*: The crystal structures of Pt/BPFMO/NSTO devices were characterized by X-ray diffraction (XRD) using Co *K*_α_ radiation (λ = 1.79 Å, smartlab 9kW, Rigaku, Japan). Synchrotron-based X-ray reflectivity (XRR) and reciprocal space mapping (RSM) were subsequently employed at the BL02U2 beamline of the Shanghai Synchrotron Radiation Facility (SSRF) to quantify in-plane/out-of-plane relaxation and chemical-induced strain. Atomic-resolution high-angle annular dark-field scanning transmission electron microscopy (HAADF-STEM) images were acquired with a spherical aberration-corrected instrument (JEOL ARM300F/GRAND-ARM, which is situated at Technical Institute of Physics and Chemistry, Chinese Academy of Sciences, Japan) at 300 kV, enabling thickness measurement and atomic arrangement analysis. Raw HAADF-STEM images were processed via the open-source HRTEM-Filter software, while fast Fourier transform (FFT) patterns were analyzed to determine phase structure. Geometric phase analysis (GPA) was implemented in Digital Micrograph software to map strain distributions by hybridizing real-space and Fourier-space data. Vector analysis of B-site elements was conducted in MATLAB to probe polarization behavior at the atomic scale. The thickness and surface morphologies of films were measured by scanning electron microscopy (GeminiSEM 300).

*Compositional and distortion characterizations*: The chemical composition of the samples was characterized by X-ray photoelectron spectroscopy (XPS, Thermo Scientific K-Alpha, USA). Semi-quantitative relative proportions of elemental chemical states in the thin films were determined through deconvolution and integration of Fe 2*p*, Mn 2*p*, and O 1*s* spectra using Avantage software. Elemental distribution and cation ordering were verified by energy-dispersive X-ray spectroscopy (EDS) mapping coupled with HAADF-STEM. Octahedral distortion and orbital hybridization within the crystal structure were validated through X-ray absorption spectroscopy (XAS) measurements conducted at the BL07U beamline of SSRF, China.

*Electrical properties characterizations*: Electrical properties characterization was performed under ambient conditions. Ferroelectric properties, including polarization-electric field (*P-E*) hysteresis loops and switching current profiles, were investigated using an aixACCT TF-Analyser 3000 system (aixACCT GmbH, Germany) operating at 10 kHz in dynamic hysteresis and PUND measurement modes. Leakage current characteristics were quantified either with a Keithley 2400 source meter (Tektronix, USA). Atomic-scale polarization behavior was probed through vector analysis of B-site elements in HAADF-STEM images, implemented via MATLAB computational routines.

*Optical properties characterizations*: Reflection spectra were acquired using a UV-Vis-NIR spectrophotometer (Shimadzu UV-2600i, Japan) with 300-800 nm spectral range. Absorption spectra were derived by subtracting reflected light intensity from incident intensity without accounting for optical loss. Photovoltaic properties were characterized via a custom system integrating a Keithley 2400 source meter (Tektronix, USA) and a four-channel LED solar simulator (NBeT, China) delivering 0-100 mW/cm^2^ irradiance under AM1.5G spectral conditions. The φ of BPFMO double perovskite thin films was determined by UPS (Thermo ESCALAB 250XI, USA), and the φ was calculated for evaluating the Schottky barrier height.

**S2 Supporting Equations**

**Equations S1.** The Bragg equation, used to calculate the out-of-plane lattice constant of the film [S1]:

2*c*sin*θ* = n*λ*

Where *c* is the crystal plane spacing, *θ* is the diffraction angle, *λ* is the X-ray wavelength and n is the multiple of crystal plane. The lattice constant out-of-plane for the STO substrate is approximately 0.3905 nm.

**Equations S2.** The thickness of the film can be calculated from the X-ray reflectivity spectrum using the following equation [S2]:

*d* = *λ*/(2Δ*θ*)

where *d* is the thickness of the thin film, *λ* is the x-ray wavelength, Δ*θ* is the period of the XRR stripes (the distance between adjacent peaks or adjacent troughs, usually calculated by averaging the values of multiple cycles.). The XRR test has strict requirements for the flatness and roughness of the surface of the film sample. Therefore, it can prove that the film material has a high-quality surface.

**Equations S3.** Chemical stress can be quantitatively evaluated through lattice deformation and elasticity of lattice. The specific method is as follows [S3]:

$$dP=-K\frac{dV}{V_{0}}$$

where *dP* is the chemical pressure, K is the bulk modulus of materials, and *dV/V_0_* is the variation of the lattice volume. As reported in the literature, the bulk modulus of

bulk BFMO double perovskite material is 114.1 GPa.

**Equations S4.** The Tauc-Lorentzian model follows the following equation when fitting the band gap [S4]:

*αhν* = A(*hν* – *E*_g_)^n^

where *hν* is the photon energy, *α* is the absorption coefficient, A is the scaling constant, *E*_g_ is the bandgap energy, and n is the transition type (n = 1/2 for direct-allowed, 3/2 for direct-forbidden, 2 for indirect-allowed transitions, and 3 for indirect-forbidden transitions respectively).

**S3 Supplementary Figures and Tables**


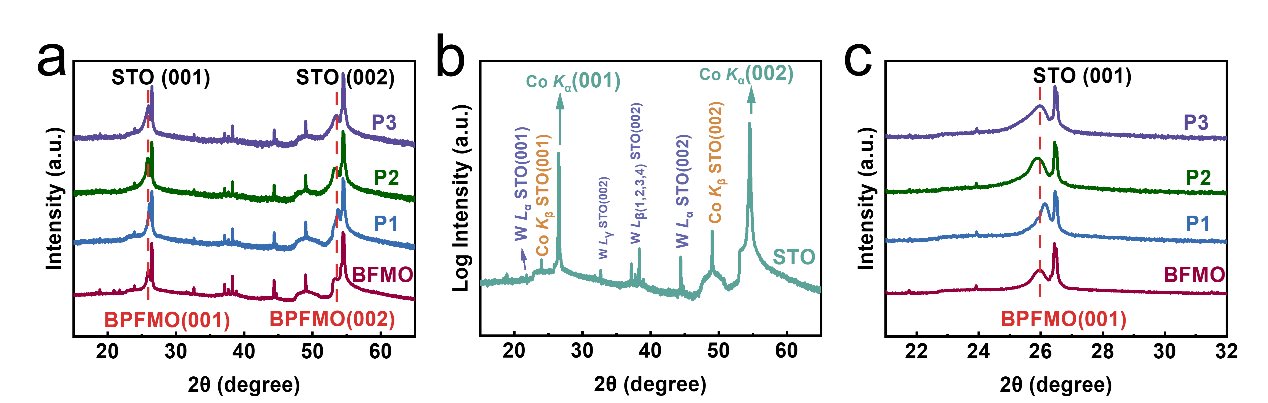


**Fig. S1** (**a**) XRD patterns of BPFMO double perovskite thin films with varying Pb stoichiometries, including the pure BFMO thin film. (**b**) XRD patterns of bare STO (001) substrate. (**c**) Partial enlarged drawing of OOP XRD diffraction patterns of P1, P2, and P3 thin film samples on STO (001) substrate


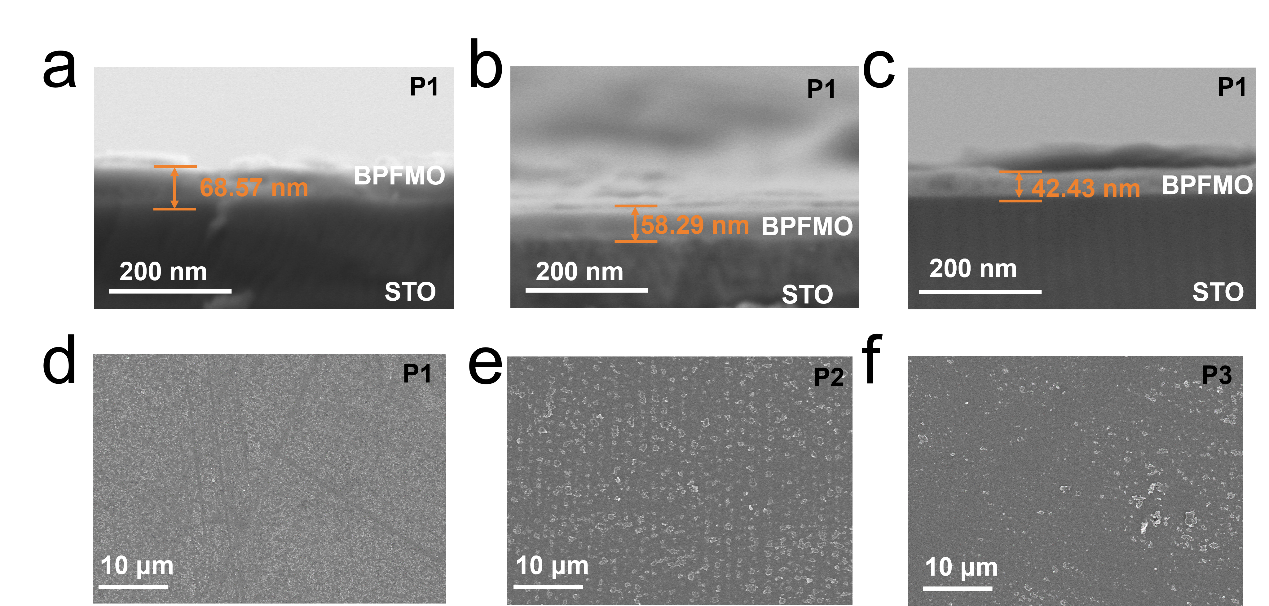


**Fig. S2** Scanning electron microscopy (SEM) images: (**a-c**) Cross-sectional views of thin films P1, P2, and P3; (**d-f**) Surface views of the corresponding thin films

**Fig. S3** EDS spectral data for the O element acquired at the P1/STO interface, alongside elemental mapping results for Bi and Pb

**Fig. S4** (**a**) Large-scale HAADF-STEM image of P1 thin film grown on STO substrate. (**b**) local-scale HAADF-STEM images of P1 double perovskite thin film on STO substrate along the STO [100] zone axis. (**c**) FFT patterns of P1 double perovskite thin film on STO substrate and (**d**) geometric phase analysis (GPA) *ε*_xx_ images (the color scale indicates different strain regions of the thin film lattice) for P1 thin film on STO substrate

**Fig. S5** XPS survey scans acquired from P1, P3, and P3 thin films


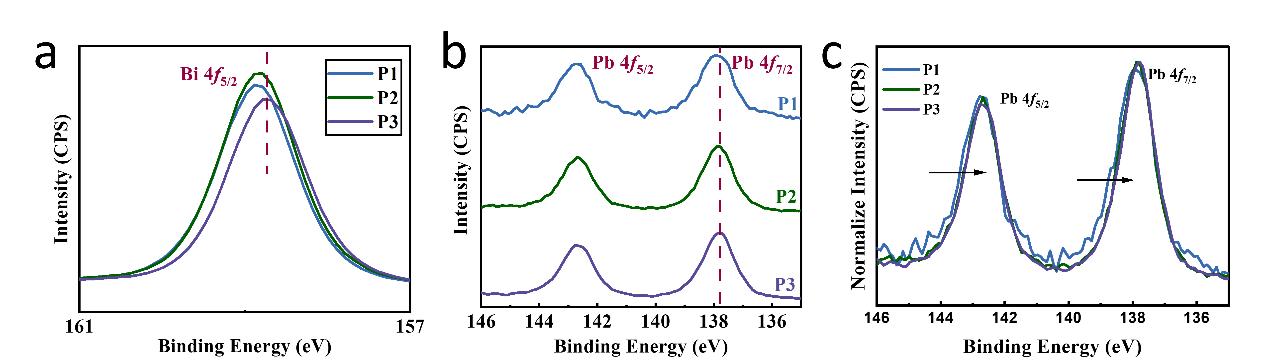


**Fig. S6** (**a**) Bi 4f XPS spectra of P1, P2 and P3 thin films. (**b**) Pb 4f XPS spectra of P1, P2 and P3 thin films. The coordinate axis scales have been normalized to facilitate observation of the peak position changes. (**c**) For comparison, the Pb 4f XPS spectra were normalized. P1 exhibits a slight peak position shift relative to P2 and P3

**Fig. S7** Similar to Fig. 2i, the Fe *L*3 edge profiles of P3 thin film are normalized and then subtracted from the normalized edge profiles of P2

**Fig. S8** Schematic of Fe-O octahedral local structures and the Fe 3*d* level split in the different site symmetries

**Fig. S9** The linear fitting of data near-zero electric field to obtain intrinsic resistivity of the BPFMO double perovskite thin films

**Fig. S10** (**a**) Fitted positive leakage current curve of P1, P2and P3 thin films through the SCLC mechanism with current density and electric field. (**b**) Fitted negative leakage current curve of P1, P2and P3 thin films through the SCLC mechanism with current density and electric field

**Fig. S11** *J*-T curves for (**a**) P1, (**b**) P2 and (**c**) P3 under on/off light conditions, lighting density ranges from 20 to 100 mW/cm^2^


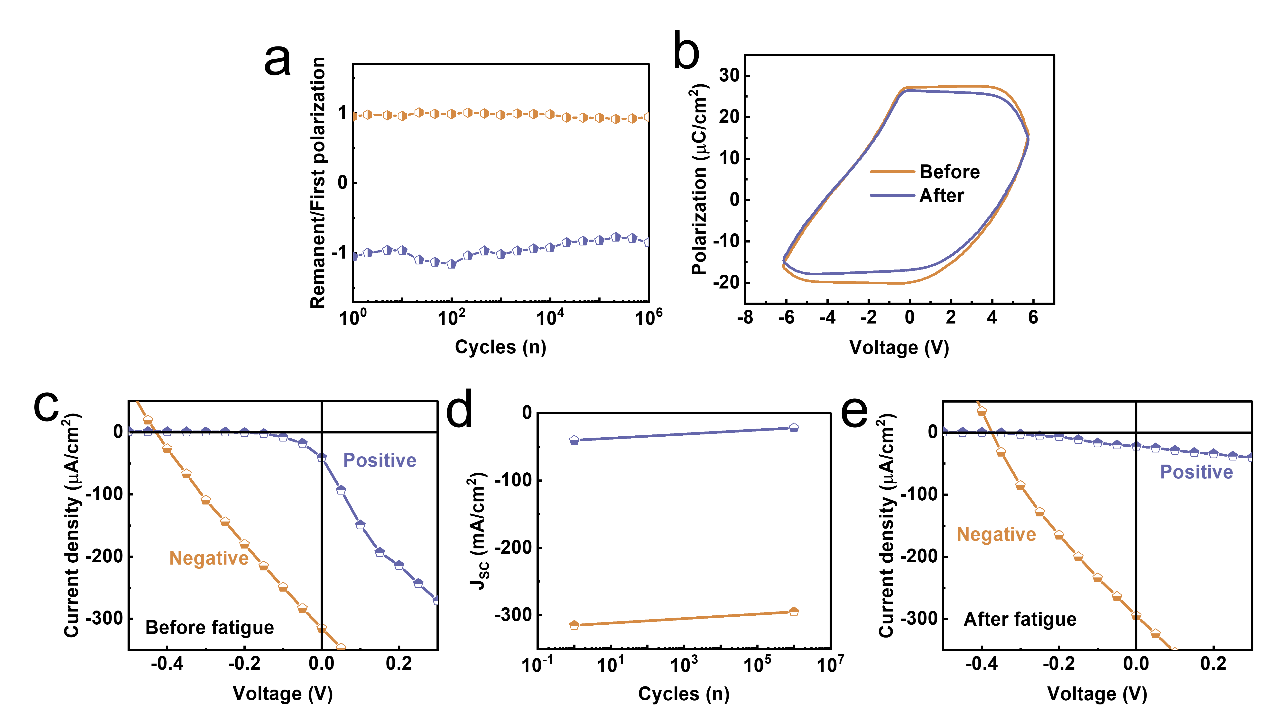


**Fig. S12** (**a**) Ferroelectric fatigue characteristics (~10^6^ times) of P1 thin films. (**b**) The hysteresis loops before and after fatigue (**c**) The Photovoltaic before fatigue with after positive and negative polarization under illumination with 80 mW/cm^2^. (**d**) Photovoltaic fatigue characteristics (~10^6^ times) of P1 thin films with after positive and negative polarization under illumination with 80 mW/cm^2^. (**e**) The Photovoltaic after fatigue with after positive and negative polarization under illumination with 80 mW/cm^2^
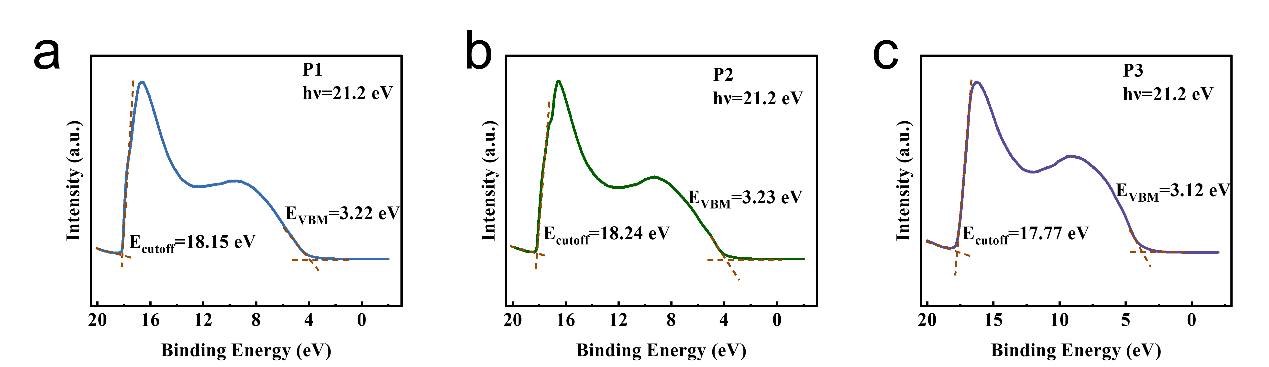


**Fig.S13** UPS spectrum of BPFMO thin films. The band alignment of the devices was investigated using ultraviolet photoelectron spectroscopy (UPS). The work functions of the P1, P2, and P3 thin films were calculated to be 3.05 eV, 2.96 eV, and 3.43 eV, respectively, by subtracting the secondary electron cutoff energy from the photon energy of the excitation radiation. The corresponding valence band maximum (VBM) positions were determined as 3.33 eV, 3.23 eV, and 3.12 eV, while the conduction band minimum (CBM) positions were found to be 1.69 eV, 1.42 eV, and 1.53 eV, relative to the vacuum level


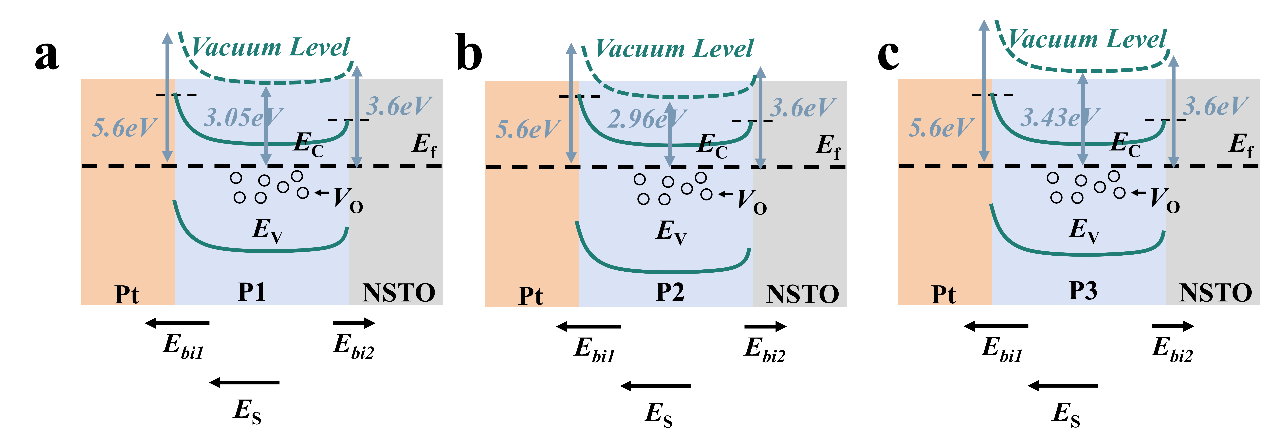


**Fig. S14** Energy band diagrams of: (**a**) Pt/P1/NSTO, (**b**) Pt/P2/NSTO, and (**c**) I Pt/P3/NSTO. *E*_V_, *E*_C_, *E*_F_, *E*_bi1_, *E*_bi2_, and *E*_S_ represent the valence band, the conduction band, the Fermi level, the built-in electric field between the Pt/thin film, the built-in electric field between the thin film/NSTO, and the start total Schottky barrier-induced field(the superposition of *E*_bi1_ and *E*_bi2_), respectively

**Table S1** The ratios of various elements in the P1, P2, and P3 thin films are calculated by integrating the fitted XPS spectral areas, and the ratios of Pb/(Bi+Pb), Mn^4+^/Mn^3+^, Fe^3+^/Fe^2+^, and O_V_/O_L_ are summarized in the table

**Table S2** The binding energies of Fe 2*p*_3/2_, Mn 2*p*_3/2_ and O 1*s* of the deconvolution fitted XPS spectra in P1, P2, and P3 thin films. The Fe 2*p*_3/2_ characteristic peaks are separated into Fe^3+^ and Fe^2+^, the Mn 2*p*_3/2_ characteristic peaks are separated into Mn^4+^and Mn^3+^, and the O 1*s* characteristic peaks are mainly concerned with the lattice oxygen (O_L_) and vacancy oxygen (O_V_)

**Table S3** The ratios of various elements in the P1, P2, and P3 thin films are calculated by integrating the fitted XPS spectral areas, each film was analyzed on its surface three times using XPS


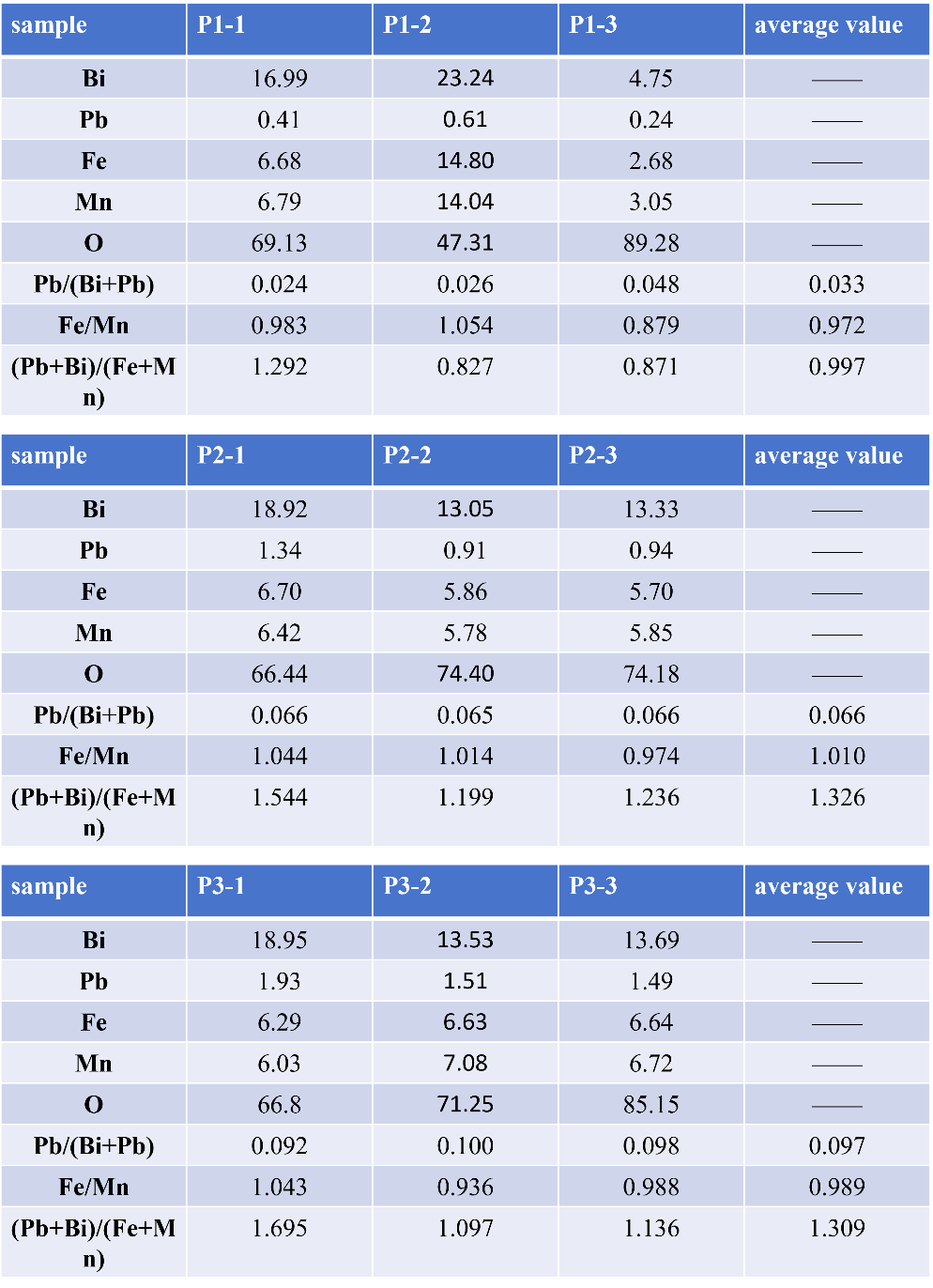


**Table S4** Results regarding the band gap and *J*s

**Supplementary References**

1. L. Zhang, J. Chen, L. Fan, O. Diéguez, J. Cao et al., Giant polarization in super-tetragonal thin films through interphase strain. Science **361**(6401), 494–497 (2018). <https://doi.org/10.1126/science.aan2433>
2. Q. Yang, J. Hu, Y.-W. Fang, Y. Jia, R. Yang et al., Ferroelectricity in layered bismuth oxide down to 1 nanometer. Science **379**(6638), 1218–1224 (2023). <https://doi.org/10.1126/science.abm5134>
3. Y. Wang, L. Zhang, J. Wang, Q. Li, H. Wang et al., Chemical-pressure-modulated BaTiO_3_ thin films with large spontaneous polarization and high curie temperature. J. Am. Chem. Soc. **143**(17), 6491–6497 (2021). <https://doi.org/10.1021/jacs.1c00605>
4. J. Tauc, Absorption edge and internal electric fields in amorphous semiconductors. Mater. Res. Bull. **5**(8), 721–729 (1970). <https://doi.org/10.1016/0025-5408(70)90112-1>
